# Patients’ and healthcare providers’ views regarding dose reduction of tyrosine kinase inhibitors in chronic myeloid leukemia: a qualitative study

**DOI:** 10.1093/oncolo/oyag114

**Published:** 2026-03-30

**Authors:** Dina N Lokhorst, Bart J F van den Bemt, Yolba Smit, Rosella P M G Hermens, Nicole M A Blijlevens, Charlotte L Bekker

**Affiliations:** Department of Hematology, Radboud University Medical Center, Nijmegen, 6525 GA, The Netherlands; Department of Pharmacy, Sint Maartenskliniek, Nijmegen, 6574 NA, The Netherlands; Department of Pharmacy, pharmacology and toxicology, Radboud University Medical Center, Nijmegen, 6525 GA, The Netherlands; Department of Hematology, Radboud University Medical Center, Nijmegen, 6525 GA, The Netherlands; IQ Health, Radboud University Medical Center, Nijmegen, 6525 GA, The Netherlands; Department of Hematology, Radboud University Medical Center, Nijmegen, 6525 GA, The Netherlands; Department of Pharmacy, pharmacology and toxicology, Radboud University Medical Center, Nijmegen, 6525 GA, The Netherlands

**Keywords:** qualitative study, dose reduction, tyrosine kinase inhibitors, chronic myeloid leukemia, COM-B

## Abstract

**Background:**

Dose reduction of tyrosine kinase inhibitors (TKIs) in patients with chronic myeloid leukaemia (CML) can reduce side effects and improve quality of life while maintaining treatment effectiveness and reducing costs.

**Objective:**

This study evaluates the views of both patients with CML and healthcare providers involved in CML care on dose reduction of TKIs.

**Methods:**

A qualitative study with semi-structured interviews via phone or video call was conducted with Dutch adult patients with CML and their healthcare providers. The COM-B model served as a theoretical framework to guide data collection and analysis to understand participants’ capability, opportunity, and motivation for dose reduction. An inductive coding approach was utilized to derive themes from the data.

**Results:**

Eighteen patients and twelve healthcare providers (haematologists, nurse specialists, and pharmacists) participated, who in general were supportive of dose reduction. Two main themes were identified: (1) willingness and acceptance of dose reduction, which was shaped by participants’ beliefs about the use of TKIs, the impact of TKIs on patients’ quality of life, and associated hopes, fears and concerns; and (2) needs and preferences regarding dose reduction such as timing, eligibility, practical aspects, and shared-decision making.

**Conclusion:**

Patients and healthcare providers have positive views on TKI dose reduction to improve the burden of CML treatment. Acceptance is shaped by their beliefs and concerns, while successful implementation depends on addressing practical needs and shared-decision making.

Implications for Practice This study explores the perspectives of patients and healthcare providers on tyrosine kinase inhibitor (TKI) dose reduction in chronic myeloid leukemia treatment. The results show that to consider dose reduction, several factors such as safety, informed and shared decision-making, healthcare resources, and patient experiences need consideration. To support this process, tools can be developed to guide both patients and providers in making informed decisions, improve understanding of side effects, and offer updated, evidence-based guidelines. These measures would enable a more personalized approach to TKI dosing, ultimately improving patient outcomes and enhancing quality of life.

## Introduction

Treatment with tyrosine kinase inhibitors (TKIs) has significantly improved chronic myeloid leukemia (CML) survival rates, allowing patients to achieve life expectancies comparable to the general population.^1^ However, many patients need lifelong treatment to attain at least a major molecular response, and long-term exposure to TKIs is associated with significant side effects affecting over half of patients.^2^ These side effects range from mild symptoms such as nausea and fatigue to more severe conditions such as myelosuppression and cardiotoxicity, which can significantly impact patients’ quality of life. To manage these side effects, numerous studies have investigated or are exploring dose optimization strategies like dose reduction and treatment discontinuation, attempting treatment free remission.^3–7^ The latter is only possible for select patients with sustained deep molecular responses and has high relapse rates (approximately 50%).^8^ Limited available evidence, primarily derived from nonrandomized studies and retrospective analyses, indicates that dose reduction has a higher success rate, with less than 24% experiencing molecular recurrence.^4–7^ These studies suggest that TKI dosages can be lowered while maintaining effectiveness, resulting in fewer side effects. This approach could also reduce the overall treatment burden and costs and improve the quality of life for patients with CML.^9^ To assess the feasibility of dose reduction, it is essential to consider the willingness of both patients and healthcare providers. Understanding both perspectives is crucial for improving CML care, as previous research identified an imbalance in the perceived impact of CML between patients and healthcare providers.^10^ Previous qualitative studies have explored patients’ views on TKI discontinuation, reporting the absence of side effects as the most important benefit, whereas fear of disease recurrence was the biggest concern.^11–14^ However, no research investigated patients’ and healthcare providers’ views towards dose reduction in CML treatment. Therefore, this study aims to identify the views of patients with CML and involved healthcare providers regarding TKI dose reduction.

## Materials and methods

### Design and setting

A qualitative study was performed by conducting semi-structured interviews with patients with CML and healthcare providers involved in CML care from multiple hospitals in the Netherlands. This study is reported according to the consolidated criteria for reporting qualitative research (COREQ) guideline to ensure explicit and comprehensive reporting (Table S1).^15^

### Ethics

Ethical review was waived by the Medical Ethical Review Committee of the Radboud University Medical Center (protocol no. 2020-7269).

### Study population

Two participant groups were identified: patients with CML and healthcare providers directly involved in CML care. Adult patients with CML (≥18 years) using a TKI and able to communicate in Dutch were recruited by their treating hematologist. Additionally, a call for participants was posted on the CML care platform (www.CMyLife.nl). Interested patients received study information by post. Healthcare providers, that is, hematologists, nurse specialists, and hospital pharmacists, were invited via standardized e-mail invitations. Purposive sampling was employed to achieve a diverse patient population that reflects Dutch CML demographics in terms of age, gender ratio, and disease duration. For healthcare providers, sampling was based on their employment in various hospital types. Informed consent was obtained from all participants before data collection.

### Theoretical framework

The Capability-Opportunity-Motivation-Behavior (COM-B) model guided the data collection and analysis, outlining three components that influence behavior: (1) capability, the individual’s physical and psychological capacity to engage in a behavior; (2) opportunity, the external social and physical factors that enable or prompt the behavior; and (3) motivation, the psychological processes—both automatic (emotions) and reflective (beliefs and intentions)—that drive participation in certain behaviors.^16^ The COM-B model is widely used in qualitative studies on healthcare interventions in oncology that require behavior change from end-users.^17–19^ Here, the model was used to understand the behavior of patients and healthcare providers regarding TKI dose reduction. Dose reduction was identified as the key behavior. Two semi-structured interview guides for patients and healthcare providers were developed, covering four main topics: (1) general views on TKI dose reduction, (2) perceived concerns about dose reduction, (3) preferences for considering dose reduction, and (4) needs for supporting decision aids for informed choices. See the supplemental file for the Needs Assessment Interview Guides. Detailed information on specific decision aid needs is shared separately by the research team.^20^

### Data collection

Semi-structured individual interviews were held by a trained female junior researcher (MD) by phone or a video call. Interviews started with an introduction of the interviewer and the study’s purpose, followed by guided questions. These conversations were audio-recorded and transcribed verbatim. During the sessions, MD took field notes. Afterwards, participants received their transcripts and could provide feedback on accuracy. Interviews were held until saturation occurred in both groups, that is, when no new information was obtained after three consecutive interviews.

### Data analysis

Inductive thematic analysis using ATLAS.ti software was performed to enable flexible data-driven discovery of themes and insights. Relevant text fragments were coded using a descriptive code. A senior researcher (C.B.) and junior researcher (N.L.) independently coded the first two transcripts. Discrepancies were discussed until consensus was reached. N.L. coded the remaining transcripts under C.B.’s supervision through audits. Similar open codes were grouped by N.L. and reviewed by C.B. to create axial codes. These codes were then organized into main and subthemes, with key factors from these themes mapped onto the COM-B model. This process was reviewed and discussed with N.L., C.B., and B.vd.B. until consensus was reached. Quotes were translated from Dutch to English.

## Results

Eighteen patients with CML and twelve healthcare providers participated (six hematologists, four nurse specialists, and two pharmacists) in on average 45-minute-long interviews. Informed consent was obtained from nineteen patients; due to data saturation, one patient was not interviewed. Healthcare providers were employed across eight hospitals in the southern and eastern regions of the Netherlands. Table 1 presents the participant characteristics.

**Table 1.** General participant characteristics.

	Patients (*n* = 19^a^)	Healthcare providers (*n* = 12)
**Gender, male (%)**	10 (52.6)	7 (58.3)
**Age, mean (SD)**	57 (9.2)	—
**Educational level (%)**		—
**High**	18 (94.7)	
**Middle**	1 (5.3)	
**Low**	—	
**Disease duration (mo), median (IQR)**	80 (120)	—
**TKI use (%)**		—
**Imatinib**	6 (31.6)	
**Dasatinib**	5 (26.3)	
**Nilotinib**	5 (26.3)	
**Bosutinib**	3 (15.8)	
**Type of hospital (%)**	—	
**Academic medical center**		3 (25)
**Top clinical**		7 (58.3)
**General hospital**		2 (16.7)

a Percentages were calculated for 19 patients.

Generally, most patients favored dose reduction and noted it as a common topic among CML patients. However, some preferred to maintain their current dosage for various reasons. Healthcare providers were generally supportive but highlighted the need for more evidence-based knowledge, as dose reduction is not a standard practice outside of managing side effects in treatment guidelines. Two main themes about patients’ and healthcare providers’ views about dose reduction in CML were identified: willingness and acceptance of dose reduction and required strategies and needs to offer dose reduction.

### Willingness and acceptance of dose reduction

Participants’ beliefs about the necessity of TKI treatment for CML and potential positive and negative consequences of dose reduction on therapy effectiveness influenced their views thereof.

#### Beliefs about TKI treatment influence the willingness for dose reduction

Patients and healthcare providers mentioned that TKIs are crucial to maintain adequate health and associated survival benefits. Some healthcare providers were thus cautious with dose reduction as they felt it was still in an experimental stage and remembered past challenges with CML before the introduction of TKIs.

*Having witnessed the dramas of this disease and knowing how challenging it can be to manage it well, I’m not about to take the lead and experiment with that.—HCP03*

*I don’t take that stuff for nothing. … I understand that you used to die with what I have. Now there is imatinib and the disease won’t kill you. That is an important difference. I take the medication because it helps.—PT13*

##### Long-term effects

Participants highlighted the potentially harmful and partly unknown long-term effects of TKIs, perceiving them as unhealthy or even as poison. They emphasized the long-term benefits of dose reduction, preferring to minimize TKI use while ensuring safety. Some viewed dose reduction as a step toward future successful treatment-free remission, often perceived as the ultimate treatment goal for patients.

*Because, however you look at it, you are still taking a dose of poison every day. If that can be reduced, it would be really great, I think.—PT14*

*TKIs definitely have an impact on people when used long-term, which I firmly believe. Hence, less is better. This is not a political statement, but fewer medications are always good.—HCP03*

##### Ecological and economic impact

Other perceived benefits of dose reduction included less environmental impact, as less drugs need to be produced, and the reduction of (societal) costs spent on CML drugs. However, neither patients nor healthcare providers considered these factors as primary drivers influencing their decision to pursue dose reduction.

#### Impact of TKI side effects on daily lives drives hopes and expectations for dose reduction

Patients reported that TKI side effects and strict intake schedules caused daily struggles and reduced their quality of life. While some acknowledged the benefits of medications outweighed some side effects, others found them highly bothersome. These challenges led to reduced will to live, insecurity, loss of spontaneity and freedom, decreased daily activity, and lower medication adherence. Patients hoped dose reductions would lessen side effects and improve energy, though many were cautious, doubting significant improvement, especially with age. Some expected short-term “withdrawal symptoms” but were willing to endure them. The experiences of participating patients and healthcare providers who underwent or performed dose reduction aligned with the hopes and concerns expressed by other participants.

*I’m quite nauseous. That is every night. … Only when you are nauseous like that you really feel like a patient. On the other hand I’m like yes, if it is life-sustaining, why are you complaining about being nauseous for one hour.—PT15*

*It rarely happens, but it sometimes happens out of frustration that I think like: those pills, now I’m tired of them. I’m not taking that thing and I’ll see what happens. That has to do with all the frustrations because you can no longer do what you want.—PT10*

*I remember the haematologist telling me that if the medication works, I could live to an old age with this condition. I thought, sure, you can say that, but I don’t want to reach 56 like this.—PT14*

*If the dosage decreases from 100 mg to 70 mg, and the side effects were to decrease by thirty percent, that would be a relief. Of course, it doesn’t work that linearly, I understand that. But any relief would be welcome.—PT12*

*Luckily, I don’t suffer much from those side effects. I actually live my life just like I always have. So, in that regard, I don’t think there are many benefits for me to gain.—PT14*

*I’ve already had one dose reduction, from 100 mg of dasatinib to 70 mg. I’ve had positive experiences with that. The side effects such as fatigue, and muscle tension and pains, have significantly decreased thereafter.—PT02*

#### Failure of dose reduction is a treatment risk causing emotional challenges

Some patients mentioned that failure on dose reduction could require a treatment switch, limiting future treatment options and endangering the opportunity for a treatment-free remission attempt. These concerns could cause fear, anxiety, and stress for patients. Hematologists further raised concerns about the risk of developing mutations after suboptimal treatment of CML, potentially causing resistance to TKI therapy.

*My goal and wish are simply to stop again… So, the most important thing now is for those levels to remain stable, enabling me to stop.—PT17*

*There’s a concern about losing effectiveness, which brings the risk of disease progression and the development of mutations. Those are the main worries.—HCP02*

### Required strategy and needs to offer dose reduction

#### Enhancing patient satisfaction with CML care through personalized dose reduction

In CML care, most dose reductions were implemented according to treatment guidelines, typically following severe side effects or unsuccessful attempts at achieving treatment-free remission. Few healthcare providers reduced doses based on patient-reported outcomes or for maintenance therapy, which are not in guidelines. Many patients were aware of the standardization of TKI dosages and advocated for a more personalized approach, particularly as they as an individuals differ from the general CML population in, for example, age. Both patients and healthcare providers highlighted the social benefits of this personalized approach; tailoring treatment to individual needs could help patients feel valued and respected by the healthcare provider, which could enhance their experience and satisfaction with care.

*That 60+ overweight woman or man, is a completely different situation than mine, yet we receive the same dose. It is understandable because it [personalised dosages] would be too labour-intensive, but that would be more patient-oriented. But I firmly believe that I might require less than someone else.—PT16*

*What I am trying to emphasize is that I am not just a medical case. I am a person, and I have many facets.—PT18*

#### A role for dose reduction after initial disease acceptance and disease stability

Patients indicated that the need for dose reduction as maintenance therapy typically emerges later in the CML journey, after overcoming initial struggles with disease acceptance and focusing on survival. As acceptance grew, new questions and needs, including the consideration for dose reduction, began to surface. Patients and healthcare providers mentioned stable blood values for a few years to be a prominent prerequisite for dose reduction, aligning with clinical practice. No other patient characteristics were identified to define eligible patients for dose reduction.

*If you had asked me this question five years ago, I would have approached this race differently. As a patient, at least from my experience, you start with a mindset that aims for a race to the bottom. So, you try to reach zero as quickly as possible. Therefore, you might think the medication can be even stronger, opting for a higher dose because that will get you to zero faster.—PT02*

#### Decision-making in dose reduction involves integrating evidence, trust, and patient preferences

##### Informed decision-making

Participants mentioned that proper information enables patients and healthcare providers to make informed decisions about dose reduction. Hematologists, supported by nurse specialists, were identified by participants to lead this process from start to follow-up. Healthcare providers needed more scientific evidence on safety before implementing dose reduction and stressed the importance of patients accurately reporting side effects, which are often underreported due to habituation, leading to underestimation of the impact of side effects on patients’ quality of life. While some patients seek information online, healthcare providers stressed the importance of accurate patient information to avoid misconceptions. Also, as patients recognized the necessity of adhering to their prescribed treatment, understanding dose reduction could be challenging. The majority supported the suggestion for the use of a patient decision aid for this decision. Both groups identified dose reduction decisions as complex, requiring a balance between medical expertise and patient preferences, necessitating a shared dialogue where hematologists have the final say.

*For patients, it’s quite challenging when they are told to take their medication daily to achieve good results for their condition, and then suddenly the dosage is halved or the medication is temporarily stopped. That is, of course, difficult for patients to handle.—HCP10*

*Clinical and real-world evidence, and the patient’s opinion, is relevant information for initiating dose reduction. You decide on something like this in a joint discussion, also because some considerations are quite subjective.—HCP03*

##### Emotional support and trust in the healthcare provider

Emotional support was considered crucial among participants alongside objective information. Personal guidance from the treating physician builds trust and facilitates sharing emotions. Many patients would perform dose reduction if their healthcare provider were to suggest it, purely because of this trusting relationship. Establishing this trust was seen as the treating professional’s responsibility.

*I have complete trust in general practitioners and specialists. They have done the studies and have been trained for this. They know what is possible and what is not. If she says it is possible, we’ll give it a try.—PT04*

#### Dose reduction implementation requires monitoring, support, and healthcare resources

After initiating dose reduction, frequent blood tests, with or without therapeutic drug monitoring, were deemed essential. This enables timely dose adjustments when blood levels would rise and reassures patients, aligning with clinical practice. However, some healthcare providers were concerned about the additional workload this will cause in CML care. Participants expressed a need for TKI tablets available in lower doses to avoid inaccuracies from breaking tablets. If unavailable, adjusting intake frequency could be an alternative solution. Patients also advocated for support with non-medical aspects after dose reduction, such as reintegration into work.

*The only thing I find important is regular check-ups. If you start reducing the dosage, you might need to do it monthly or every two months. This ensures better monitoring of your values. This way, you can quickly adjust if things go in the wrong direction.—PT10*

*Research into dose reduction cannot be separated from its societal context. Some people had to sell their businesses; others lost their jobs. But how does a registered cancer patient, dismissed due to their illness, find employment again once they feel better with a lower dosage?—PT18*

### Views on dose reduction mapped to the COM-B model

**Figure 1.**
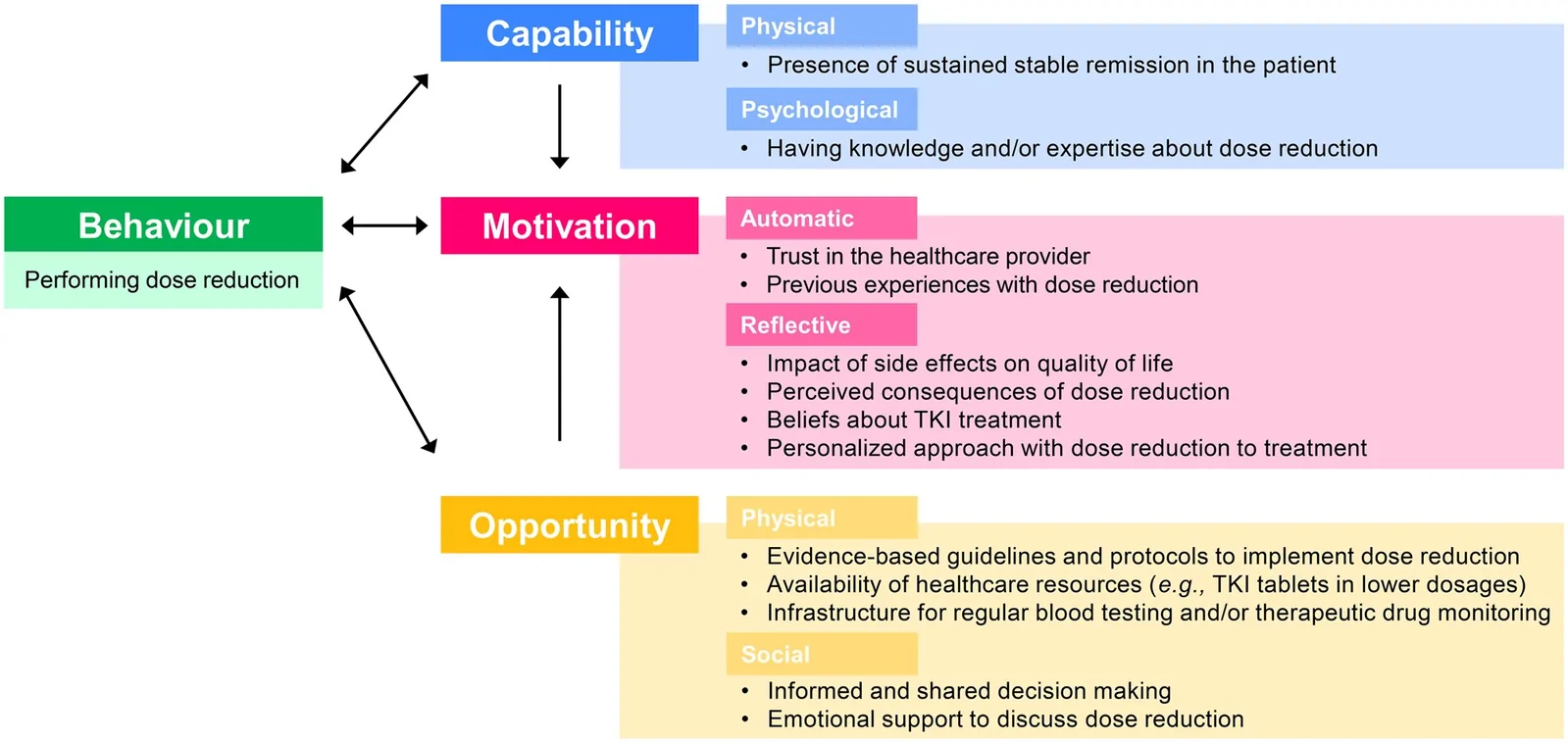
Key factors from themes identified by patients and healthcare providers for performing dose reduction mapped to the COM-B model.

Key factors from the identified themes were mapped to the COM-B model to understand dose reduction (see Figure 1).

The willingness of patients and healthcare providers to engage in dose reduction is shaped by their capabilities, motivation, and opportunities. Dose reduction itself also influenced these factors, creating a dynamic relationship. Capability factors included stable disease remission in the patient. Clinical knowledge and expertise about dose reduction enhance the psychological capability of both parties to consider dose reduction. Opportunity factors included evidence-based guidelines and protocols for efficient implementation and adequate healthcare resources. Additionally, a supportive network provides valuable perspectives and emotional support for discussions about dose reduction. Automatic motivation was influenced by trust in the healthcare provider and previous experiences with dose reduction, and reflective motivation was influenced by side effects, perceived outcomes of dose reduction, and personal beliefs about the medication.

## Discussion

This study identified two primary themes on TKI dose reduction in CML treatment from the perspectives of patients and healthcare providers. The first theme explored the willingness and acceptance of dose reduction, which was shaped by beliefs about TKI therapy, the impact of medication on quality of life, and participants’ hopes and concerns. While patients recognized the importance of TKIs for their health, they felt that these side effects diminished their quality of life and motivated them for dose reduction. However, their willingness to reduce doses was challenged by concerns about potential risks, including fear of disease recurrence. Healthcare providers also supported dose reduction and shared similar concerns. Particularly hematologists were more conservative due to past experiences and concerns about treatment resistance. The second theme focused on strategies and needs required to facilitate dose reduction. Both patients and healthcare providers advocated for more personalized dosing once stable blood levels were achieved, provided frequent blood monitoring is conducted. They believed that tailoring dosages to individual needs would improve patient outcomes and quality of life. Healthcare providers emphasized the need for further research on the potential risks and benefits of dose reduction. A shared decision-making process, based on trust and aligning medical decisions with patients’ preferences, was considered crucial, with many supporting a patient decision aid to guide this choice.

The study mapped key factors to the COM-B model, which emphasizes that TKI dose reduction relies on the interaction of patients’ and healthcare providers’ capabilities, motivation, and opportunities. Effective dose reduction strategies must therefore address all three components. This can be achieved through tools that facilitate informed decision-making, improve understanding of side effects, and provide updated, evidence-based guidelines. Integrating guidance for dose reduction into CML treatment guidelines is particularly important, as it enables clinicians to make confident decisions without perceiving the approach as experimental. When combined with tools to support SDM, such as patient decision aids, patients can be actively involved in the decision-making process, ensuring that their values and needs are fully considered. In this context, our team has already developed and alpha-tested a patient decision aid to support CML patients considering TKI dose reduction as part of the ongoing prospective, multicenter RODEO trial (EudraCT: 2021-006581-20, https://doi.org/10.1186/s12885-023-10697-6).^3^^,^^20^ Additionally, tools that improve the recognition and understanding of side effects can enable both healthcare providers and patients to better assess their burden and take appropriate action. By addressing the common issues of underreporting and underestimation, such tools have the potential to enhance clinical management and ultimately improve patients’ quality of life.

Although no prior studies have specifically explored both patient and healthcare provider views on TKI dose reduction, these findings align with research on patients’ willingness to discontinue treatment.^11–14^ Similar concerns, particularly fear of disease recurrence, were common, while reducing side effects was a key motivator. Patient education and trust in healthcare providers emerged as recurring themes in other qualitative studies on tapering for chronic diseases, highlighting the essential role of shared decision-making in achieving informed, individualized treatment choices.^21–23^ Furthermore, recent results from the multinational CML SUN study, which investigated unmet needs among patients with chronic-phase CML and their physicians, reinforce the importance of patient-centered approaches, particularly through SDM and patient education.^24^

Combined with effectiveness evaluation trials, these findings suggest that dose reduction could be an acceptable and feasible option to reduce the burden of side effects and long-term toxicities.^4–7^ However, earlier studies used strict selection criteria and standardized reductions, while many patients and healthcare providers advocated for a personalized approach to dose reduction. The RODEO trial specifically addresses this by using a patient decision aid and shared decision-making to guide personalized TKI dose reductions in a real-world setting.^3^ Shared decision-making encourages collaboration between clinician and patient, ensuring dose reduction decisions are guided by both medical information and the patient’s preferences and life goals.^25^ As many patients view treatment discontinuation as an ultimate goal, future studies should consider dose reduction as a potential step towards that goal, while addressing the concerns and challenges highlighted in this study.

The major strength of this study is the exploration of both patients’ and healthcare providers’ views on dose reduction, which is essential for improving CML care.^10^ Integrating both perspectives helps guide a more effective approach to implementing dose reduction. However, several limitations were noted. The study may have suffered from selection bias, as more highly educated patients participated, potentially skewing the results. In addition, the use of video interviews limited the observation of non-verbal communication. Nonetheless, this approach was considered, as it was more convenient to participants and facilitates a safe environment for open conversation. Unfortunately, quotes could not be linked to the specific participant, providing limited context.

## Conclusion

To conclude, patients’ and healthcare providers’ views on dose reduction of TKIs in the treatment of CML are influenced by the willingness and acceptance of dose reduction, which is shaped by their beliefs about TKI treatment, the impact of TKIs on patients’ quality of life, and their fears and concerns. Furthermore, when offering dose reduction, several factors must be covered, including timing, eligibility, practical aspects, and decision-making processes.

## Supplementary Material

oyag114_Supplementary_Data

## Data Availability

The data supporting the findings of the study can be made available from the corresponding author upon reasonable request.
