# Correction to: Patients’ and healthcare providers’ views regarding dose reduction of tyrosine kinase inhibitors in chronic myeloid leukemia: a qualitative study

**DOI:** 10.1093/oncolo/oyag442

**Published:** 2026-11-13

This is a correction to: Dina N Lokhorst, Bart J F van den Bemt, Yolba Smit, Rosella P M G Hermens, Nicole M A Blijlevens, Charlotte L Bekker, Patients’ and healthcare providers’ views regarding dose reduction of tyrosine kinase inhibitors in chronic myeloid leukemia: a qualitative study, *The Oncologist*, Volume 31, Issue 5, May 2026, oyag114, https://doi.org/10.1093/oncolo/oyag114

In the originally published version of the paper, Dina Lokhorst was marked to be the corresponding author. This was changed and Charlotte L. Bekker is now corresponding author on this article.

