# Supplementary material for: Patients’ and healthcare providers’ views regarding dose reduction of tyrosine kinase inhibitors in chronic myeloid leukemia: a qualitative study
Source: Oncologist. 2026 Mar 30;31(5):oyag114. doi: 10.1093/oncolo/oyag114 (PMC13071400; doi:10.1093/oncolo/oyag114)
Supplement: oyag114_Supplementary_Data [file oyag114_supplementary_data.docx]

# Supplementary Table S1 – Consolidated criteria for reporting qualitative studies (COREQ): 32-item checklist

| No | Item | Guide questions/description | Described in section |
| --- | --- | --- | --- |
| Domain 1: Research team and reflexivity | | | |
| Personal Characteristics | | | |
| 1. | Interviewer/facilitator | Which author/s conducted the interview or focus group? | Data collection |
| 2. | Credentials | What were the researcher's credentials? E.g. PhD, MD | - |
| 3. | Occupation | What was their occupation at the time of the study? | Data collection |
| 4. | Gender | Was the researcher male or female? | Data collection Data analysis |
| 5. | Experience and training | What experience or training did the researcher have? | Data collection Data analysis |
| Relationship with participants | | | |
| 6. | Relationship established | Was a relationship established prior to study commencement? | - |
| 7. | Participant knowledge of the interviewer | What did the participants know about the researcher? e.g. personal goals, reasons for doing the research | Data collection |
| 8. | Interviewer characteristics | What characteristics were reported about the interviewer/facilitator? e.g. Bias, assumptions, reasons and interests in the research topic | Data collection |
| Domain 2: study design | | | |
| Theoretical framework | | | |
| 9. | Methodological orientation and Theory | What methodological orientation was stated to underpin the study? e.g. grounded theory, discourse analysis, ethnography, phenomenology, content analysis | Theoretical framework |
| Participant selection | | | |
| 10. | Sampling | How were participants selected? e.g. purposive, convenience, consecutive, snowball | Study population |
| 11. | Method of approach | How were participants approached? e.g. face-to-face, telephone, mail, email | Study population |
| 12. | Sample size | How many participants were in the study? | Results |
| 13. | Non-participation | How many people refused to participate or dropped out? Reasons? | N/A |
| Setting | | | |
| 14. | Setting of data collection | Where was the data collected? e.g. home, clinic, workplace | Design and setting |
| 15. | Presence of non-participants | Was anyone else present besides the participants and researchers? | Data collection |
| 16. | Description of sample | What are the important characteristics of the sample? e.g. demographic data, date | Results |
| Data collection | | | |
| 17. | Interview guide | Were questions, prompts, guides provided by the authors? Was it pilot tested? | S2 |
| 18. | Repeat interviews | Were repeat interviews carried out? If yes, how many? | N/A |
| 19. | Audio/visual recording | Did the research use audio or visual recording to collect the data? | Data collection |
| 20. | Field notes | Were field notes made during and/or after the interview or focus group? | Data collection |
| 21. | Duration | What was the duration of the interviews or focus group? | Results |
| 22. | Data saturation | Was data saturation discussed? | Data collection |
| 23. | Transcripts returned | Were transcripts returned to participants for comment and/or correction? | Data collection |
| Domain 3: analysis and findings | | | |
| Data analysis | | | |
| 24. | Number of data coders | How many data coders coded the data? | Data analysis |
| 25. | Description of the coding tree | Did authors provide a description of the coding tree? | N/A |
| 26. | Derivation of themes | Were themes identified in advance or derived from the data? | Data analysis |
| 27. | Software | What software, if applicable, was used to manage the data? | Data analysis |
| 28. | Participant checking | Did participants provide feedback on the findings? | N/A |
| Reporting | | | |
| 29. | Quotations presented | Were participant quotations presented to illustrate the themes / findings? Was each quotation identified? e.g. participant number | Results |
| 30. | Data and findings consistent | Was there consistency between the data presented and the findings? | Results |
| 31. | Clarity of major themes | Were major themes clearly presented in the findings? | Results |
| 32. | Clarity of minor themes | Is there a description of diverse cases or discussion of minor themes? | Results |

# Supplementary 2 – Needs Assessment Interview Guides

1. *Interview guide patient*

General view (attitude)

1. What do you know about dose reduction of anticancer medication?
2. How do you feel about reducing the dose of your anticancer medication?
3. When do you think dose reduction might be possible?

Perceived risks and benefits

1. What do you think would happen if your anticancer medication were reduced?
2. What do you think potential benefits could be with dose reduction?
3. What do you think potential risks could be with dose reduction?
4. What is a reason for you to (not) reduce/continue?
   1. What do you expect with (fewer side effects/changed disease status/reverting dose)?
5. Which reason(s) is most important to you?
6. To what extent do you think it is possible for you to reduce/how likely do you consider it?

Needs and preferences

1. What do you need to make this decision?
2. What do you expect from your healthcare provider when making this decision?
3. Who has the decision-making authority regarding dose reduction (patient/doctor)? Why?
4. How would you like to be informed about possible dose reduction?

Need for supporting decision aids

1. What do you think of this?
2. What experience do you have with using a decision aid?
3. Would you use a decision aid if it were available?
   1. What are reasons for you to use a decision aid?
   2. What are reasons for you not to use a decision aid?
4. What do you expect from the decision aid?
5. How should the decision aid look?
   1. Format: online/paper/other..?
   2. What information should definitely be included in the decision aid?
6. By whom should the decision aid be offered?
7. *Interview guide healthcare provider*

General view (attitude)

1. What do you know about dose reduction of TKIs?
2. How do you feel about dose reduction of TKIs? To what extent do you apply this to your patients?
3. When do you think dose reduction might be possible? For example, disease timeline, patient characteristics?

Perceived risks and benefits

1. What do you think potential benefits could be with dose reduction?
2. What do you think potential risks could be with dose reduction?
   1. What is needed to mitigate these risks?
3. What is a reason for you to (not) reduce the dose?
   1. Which reason weighs most heavily for you?

Preferences

1. What do you need to apply/facilitate dose reduction with TKIs? a. For example, evidence on effectiveness, policy adjustments, etc.
2. What is the role of the patient in the decision-making process?
3. What is your role in the decision-making process?
4. Who has the decision-making authority regarding dose reduction (patient/doctor/pharmacist/nurse)? Why? How do you envision that?
5. According to you, which other parties are involved, and what roles do they play?

Need for supporting decision aids

1. What do you think of this?
2. What experience do you have with using a decision aid?
3. Would you use a decision aid for dose reduction if it were available? What are reasons for you to use a decision aid? What are reasons for you not to use a decision aid?
4. At what stage of treatment would you use it?
5. How would you use it? For example, during consultations, as assistance with patient questions.
6. Do you think there should be a separate decision aid for healthcare providers and patients? Why (not)?
7. What do you expect from the decision aid?
8. What information should definitely be included in the decision aid(s)? What are the pros/cons of dose reduction? Would you like to see images/videos in the decision tree? If yes, which ones?

How should the information be delivered? (paper/online/?)
